# Cholesterol depletion by methyl-β-cyclodextrin augments tamoxifen induced cell death by enhancing its uptake in melanoma

**DOI:** 10.1186/1476-4598-13-204

**Published:** 2014-09-01

**Authors:** Naoshad Mohammad, Parmanand Malvi, Avtar Singh Meena, Shivendra Vikram Singh, Balkrishna Chaube, Garikapati Vannuruswamy, Mahesh J Kulkarni, Manoj Kumar Bhat

**Affiliations:** National Centre for Cell Science, NCCS Complex, Ganeshkhind, Pune, 411007 India; Proteomics Facility, Division of Biochemical Sciences, National Chemical Laboratory, Pune, 411008 India

**Keywords:** Tamoxifen, Methyl β-cyclodextrin, Cholesterol, Caveolin-1

## Abstract

**Background:**

Despite modern advances in treatment, skin cancer is still one of the most common causes of death in the western countries. Chemotherapy plays an important role in melanoma management. Tamoxifen has been used either alone or in- combination with other chemotherapeutic agents to treat melanoma. However, response rate of tamoxifen as a single agent has been comparatively low. In the present study, we investigated whether treatment with methyl-β-cyclodextrin (MCD), a cholesterol depleting agent, increases the efficacy of tamoxifen in melanoma cells.

**Methods:**

This was a two-part study that incorporated *in vitro* effects of tamoxifen and MCD combination by analyzing cell survival, apoptosis and cell cycle analysis and *in vivo* antitumor efficacy on tumor isografts in C57BL/6J mice.

**Results:**

MCD potentiated tamoxifen induced anticancer effects by causing cell cycle arrest and induction of apoptosis. Sensitization to tamoxifen was associated with down regulation of antiapoptotic protein Bcl-2_,_ up-regulation of proapoptotic protein Bax, reduced caveolin-1 (Cav-1) and decreased pAkt/pERK levels. Co-administration of tamoxifen and MCD caused significant reduction in tumor volume and tumor weight in mice due to enhancement of drug uptake in the tumor. Supplementation with cholesterol abrogated combined effect of tamoxifen and MCD.

**Conclusion:**

Our results emphasize a potential synergistic effect of tamoxifen with MCD, and therefore, may provide a unique therapeutic window for improvement in melanoma treatment.

**Electronic supplementary material:**

The online version of this article (doi:10.1186/1476-4598-13-204) contains supplementary material, which is available to authorized users.

## Background

Melanoma, a dreaded form of skin cancer, affects deeper skin layers of the body and spreads rapidly to other tissues and organs [1]. Chronic exposure of skin to sunlight or UV radiation is a major risk factor for the development of melanoma characterized by alterations in the synthesis of melanin pigment [2, 3]. Chemotherapeutic drugs, radiation and immunotherapy have been widely used to treat melanoma [4, 5]. In spite of these options, the median survival rate of melanoma patients is approximately 6 months and hardly 5% of these patients may survive upto 5 years [6]. Tamoxifen (TAM) is a key member of the selective estrogen receptor modulator family used for the treatment of breast cancer, glioma, cholangiocarcinoma, ovarian cancer and leukemia [7–11]. Tamoxifen inhibits estrogen receptor (ER), although it may exert its effect in ER independent manner too. While the expression of ER in melanoma remains ambiguous, the anticancer effect of tamoxifen has been studied in various melanoma cells [12–14]. Tamoxifen in- combination with other drugs has shown marginal success in combating melanoma [15, 16]. Failures are attributed to the development of resistance due to limited drug distribution within the tumor cells. Therefore, we hypothesized that increase in the levels of drugs into tumor cells may eventually enhance the therapeutic index.

Plasma membrane contains nanometer-sized dynamic microdomains enriched in cholesterol, sphingolipids and gangliosides. These microdomain structures are integral to the regulation of influx or efflux of drugs. Depletion of membrane cholesterol disrupts integrity of lipid rafts and concurrently enhances the permeability of ions and small non-electrolytes [17, 18]. Among various cholesterol depleting agents available, methyl-β-cyclodextrin (MCD), a highly water soluble cyclic heptasaccharide consisting of β- [1–4] glucopyranose unit, is the most effective agent for depletion of cholesterol from the cells [19, 20]. We and others have shown that MCD or its modified forms enhance the cytotoxic effect of various chemotherapeutic drugs [21–23].

We demonstrate that in comparison to tamoxifen alone, MCD treatment enhances the sensitivity of cells towards tamoxifen and thus establishes a promising new strategy for improvement in the outcome of chemotherapy.

## Results

### Methyl-β-cyclodextrin enhances the susceptibility of A375 and B16F10 cells to tamoxifen

Tamoxifen treatment causes a dose-dependent decrease in the survival of A375, B16F10 and B16F1 cells and IC_50_ was calculated to be 30 μM, 40 μM and 30 μM respectively (Additional file 1: Table S1). We checked the effect of MCD by treating A375 and B16F10 cells with 2.5 mM of MCD for 1 h or 4 h and no significant change in cell cycle progression, cell survival and LDH release was observed at 2.5 mM dose of MCD for 4 h. MCD at 5 mM concentration was toxic (Additional file 2: Figure S1) and similar results were also obtained in B16F1 cells (Additional file 3: Figure S2A) by cell survival assay. For all subsequent experiments, 2.5 mM MCD was used.

**Figure 1:**
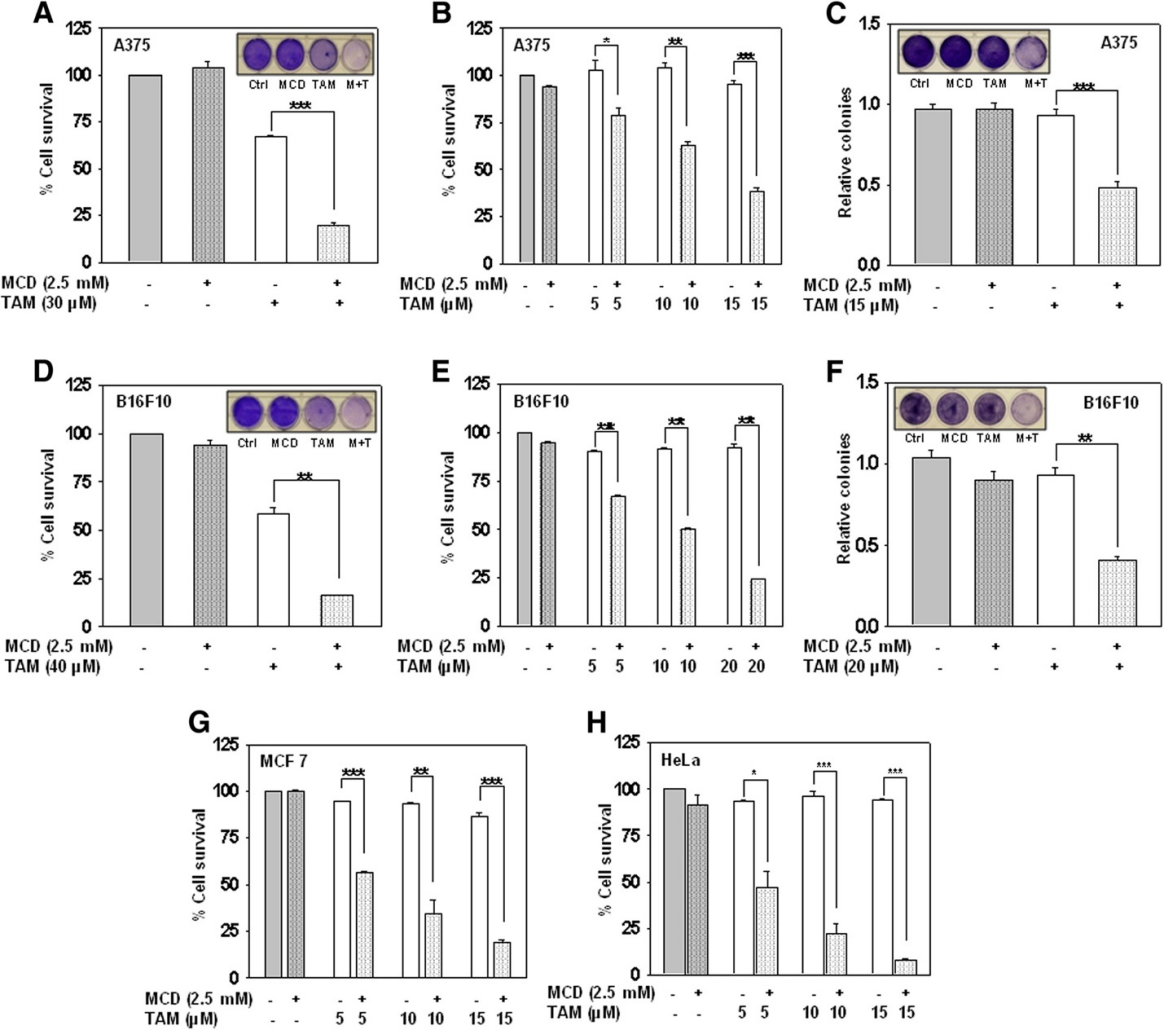
**MCD augments antiproliferative activity of tamoxifen on tumor cells** ***in vitro*** **.** Combination of tamoxifen and MCD exerts synergistic effect on different cancer cell lines. **(A-B)** A375 and **(D-E)** B16F10 cells were treated with indicated concentrations of tamoxifen together with MCD for 24 h. Data were presented as percentage of cell proliferation measured by MTT assay. **(C and F)** A375 and B16F10 cells respectively were treated with indicated concentration of tamoxifen along with MCD. Long term clonogenic survival assay was performed and colonies were assessed by Image J software. **(G and H)** MCF-7 and HeLa cells respectively were treated with indicated concentrations of tamoxifen together with MCD for 24 h. Data were presented as percentage of cell proliferation measured by MTT assay. Bar graph represents the Mean ± SD of an experiment done in triplicate (*P ≤ 0.05, **P ≤ 0.001, ***P ≤ 0.0001).

We screened the effect of various chemotherapeutic drugs like carboplatin (Carb), 5-fluorouracil (5-FU), doxorubicin (DOX), tamoxifen (TAM) and dacarbazine (DTIC) in-combintaion with MCD in A375 and B16F10 cells. While MCD specifically enhanced the cytotoxicity of tamoxifen and DTIC, it did not significantly enhance cell death induced by Carb, 5-FU and DOX (Additional file 4: Figure S3) (Additional file 5: Figure S4) in A375 and B16F10 cells. Although, DTIC is the preferred drug in melanoma treatment, which is used at 5–10 times the dose of tamoxifen [12], it is desirable to define a strategy to enhance the therapeutic efficacy of tamoxifen, a well known ER antagonist. We investigated the combinatorial effect of IC_50_ concentration of tamoxifen with MCD. Expectedly, at IC_50_ concentration of tamoxifen, cell survival diminished by 50%, which reduced to ≤35% (A375) and 40% (B16F10) in the presence of MCD and these results are clearly seen in long term clonogenic assay (Figure 1A, D). Combination of tamoxifen and MCD not only inhibited the proliferation of melanoma cells (A375 and B16F10) but also other human cell lines, viz., MCF-7 (breast cancer cell line) and HeLa (cervical cancer cell line) in a tamoxifen dose dependent manner (Figure 1B, E and G, H). To determine the long term effect of tamoxifen and MCD combination by clonogenic cell survival assay, cells were treated for 24 h with both drugs together or either agent alone and allowed to grow for 10–15 days. Number of colonies was reduced significantly in- combination treatment as compared to either agent alone (Figure 1C, F). LDH release assay also confirms that MCD enhanced the growth inhibitory effect of tamoxifen (Additional file 2: Figure S1). In addition, we found that coefficient of drug interaction (CDI) was less than 0.9 for A375 and B16F10 cells, suggesting that tamoxifen and MCD combination synergistically inhibited proliferation of A375 and B16F10 cells. (Additional file 1: Table S2). On the contrary, no significant alteration in cell survival and changes in the number of colonies were observed in B16F1 cells (non-metastatic) treated with tamoxifen and MCD combination (Additional file 3: Figure S2).

### Methyl-β-cyclodextrin enhances tamoxifen induced cytotoxicity through cell cycle arrest and induction of apoptosis

**Figure 2:**
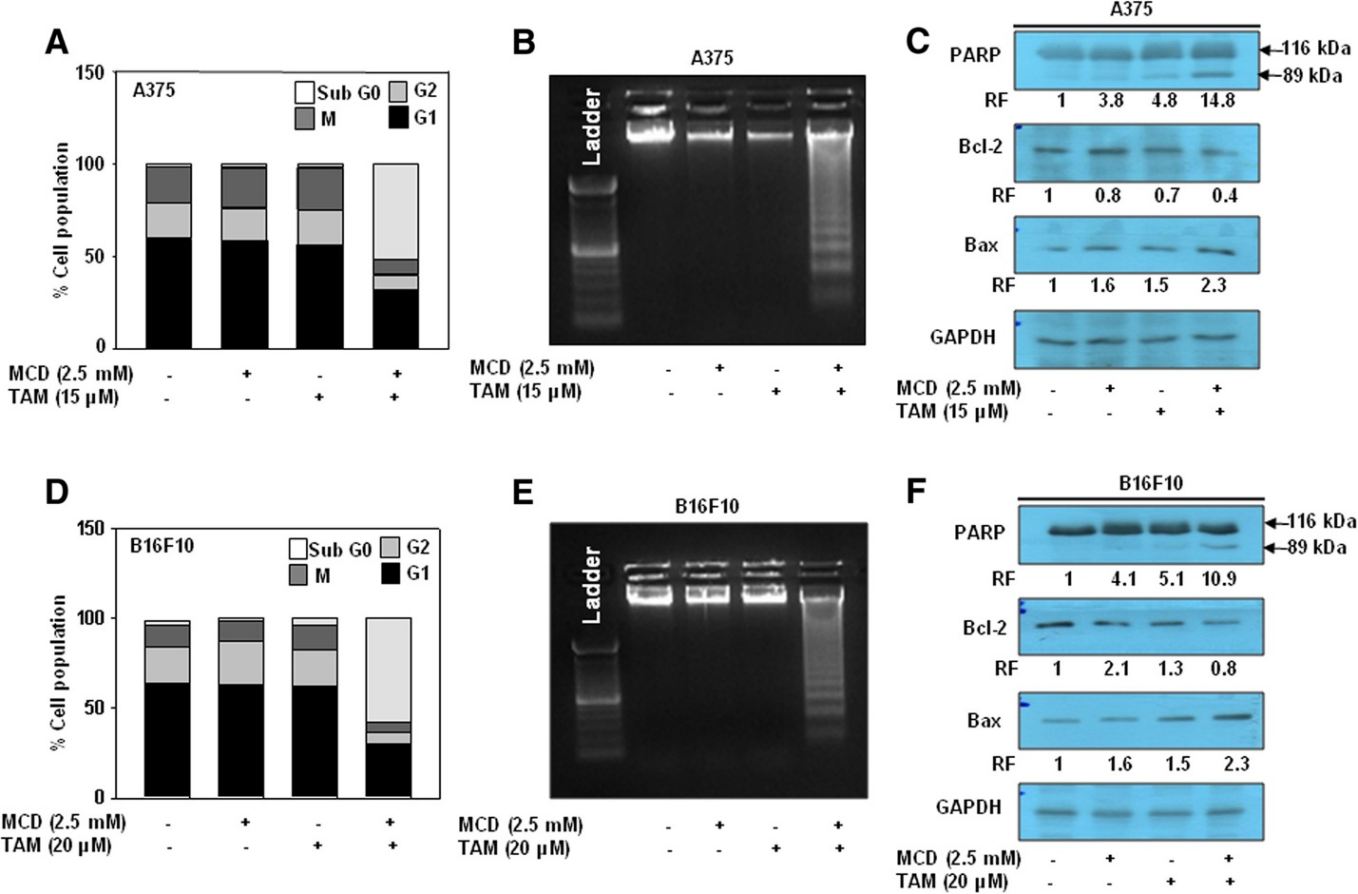
**MCD enhances the susceptibility of A375 and B16F10 cells to tamoxifen by alteration in cell cycle and DNA fragmentation.** A375 and B16F10 cells were treated with indicated concentration of tamoxifen along with MCD. **(A and D)** Display of the percentage cells in different phases of cell cycle by flow cytometry. **(B and E)** The treated cells were assayed by DNA fragmentation. **(C and F)** Whole cell lysate were subjected to Western blotting and expression level of PARP, Bcl-2 and Bax was detected. GAPDH served as a loading control. Relative folds (RF) values are expressed in arbitrary unit. Data are representative of experiments performed in triplicate.

Since tamoxifen in-combination with MCD influenced cell survival, we performed cell cycle analysis and apoptosis related experiments to further characterize synergistic effect in A375 and B16F10 cells. When cells were treated with tamoxifen and MCD combination, the percentage of cells in Sub G_0_/G_1_ phase increased dramatically to ≥50% with concomitant reduction in the population of cells in other phases as compared to either agent alone (Figure 2A, D). Further, we investigated the status of cell cycle regulatory proteins, estrogen receptor alpha (ERα) and estrogen receptor beta (ERβ) by Western blot analysis. In cells treated with tamoxifen and MCD combination, significant reduction in the levels of cell cycle regulatory proteins pRb, cyclin D1, CDK1/2, CDK4 and ERα and up-regulation of ERβ was detected (Additional file 6: Figure S5). Reduced cell survival is a consequence of apoptotic death in cells treated with tamoxifen and MCD combination as evident by detection of enhanced internucleosomal cleavage of DNA (Figure 2B, E), induction of PARP cleavage, up-regulation of Bax and down regulation of Bcl-2 protein levels in A375 and B16F10 cells (Figure 2C, F).

### Cholesterol supplementation rescues methyl-β-cyclodextrin enhanced susceptibility of cells towards tamoxifen

**Figure 3:**
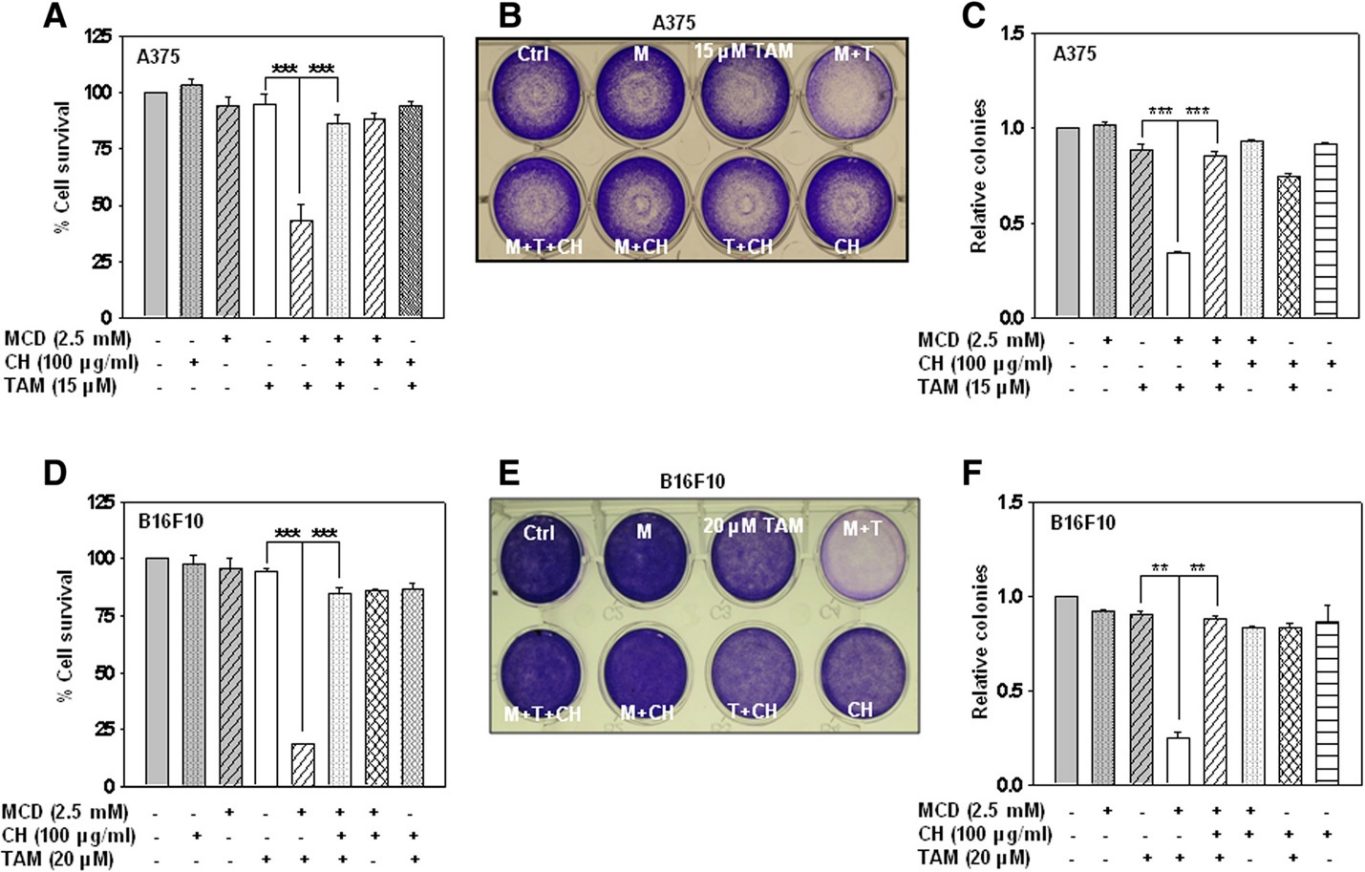
**MCD treatment enhances the susceptibility of cells to tamoxifen induced cell death and cholesterol treatment reverses the effect. (A and D)** A375 and B16F10 cells were treated with indicated concentration of tamoxifen in- combination with MCD and cholesterol. Data were presented as percentage of cell proliferation measured by MTT assay. **(B and E)** Long term clonogenic survival assay. **(C and F)** Relative quantitation of colonies by Image J software in different fields. Bar graph in MTT assay represents the Mean ± SD of experiments done multiple times in triplicate (*P ≤ 0.05, **P ≤ 0.001, ***P ≤ 0.0001).

To correlate depletion of membrane cholesterol with enhanced cytotoxic effect of tamoxifen, A375 and B16F10 cells were exposed to tamoxifen and MCD in the presence or absence of cholesterol (100 μg/ml) supplemented directly in the medium and cell survival was assessed by MTT assay. Survival was reduced by ≥50% in cells treated with tamoxifen and MCD combination. Tamoxifen induced MCD potentiated cell death was completely abrogated in cells cultured in cholesterol supplemented medium (Figure 3A, D). Also, in cells supplemented with cholesterol, the number of surviving colonies was similar to untreated cells (Figure 3B, E and C, F). Total cellular cholesterol level was reduced by >25% in the cells treated with tamoxifen and MCD combination as compared to tamoxifen alone (Additional file 7: Figure S6). Normalization of cholesterol level in MCD treated cells was achieved by culturing cells in cholesterol rich medium (Additional file 7: Figure S6). Similarly, in mice fed with 2% cholesterol, serum cholesterol level was elevated by day 15 to day 30 (data not shown).

### Co-administration of methyl-β-cyclodextrin and tamoxifen retards melanoma progression

**Figure 4:**
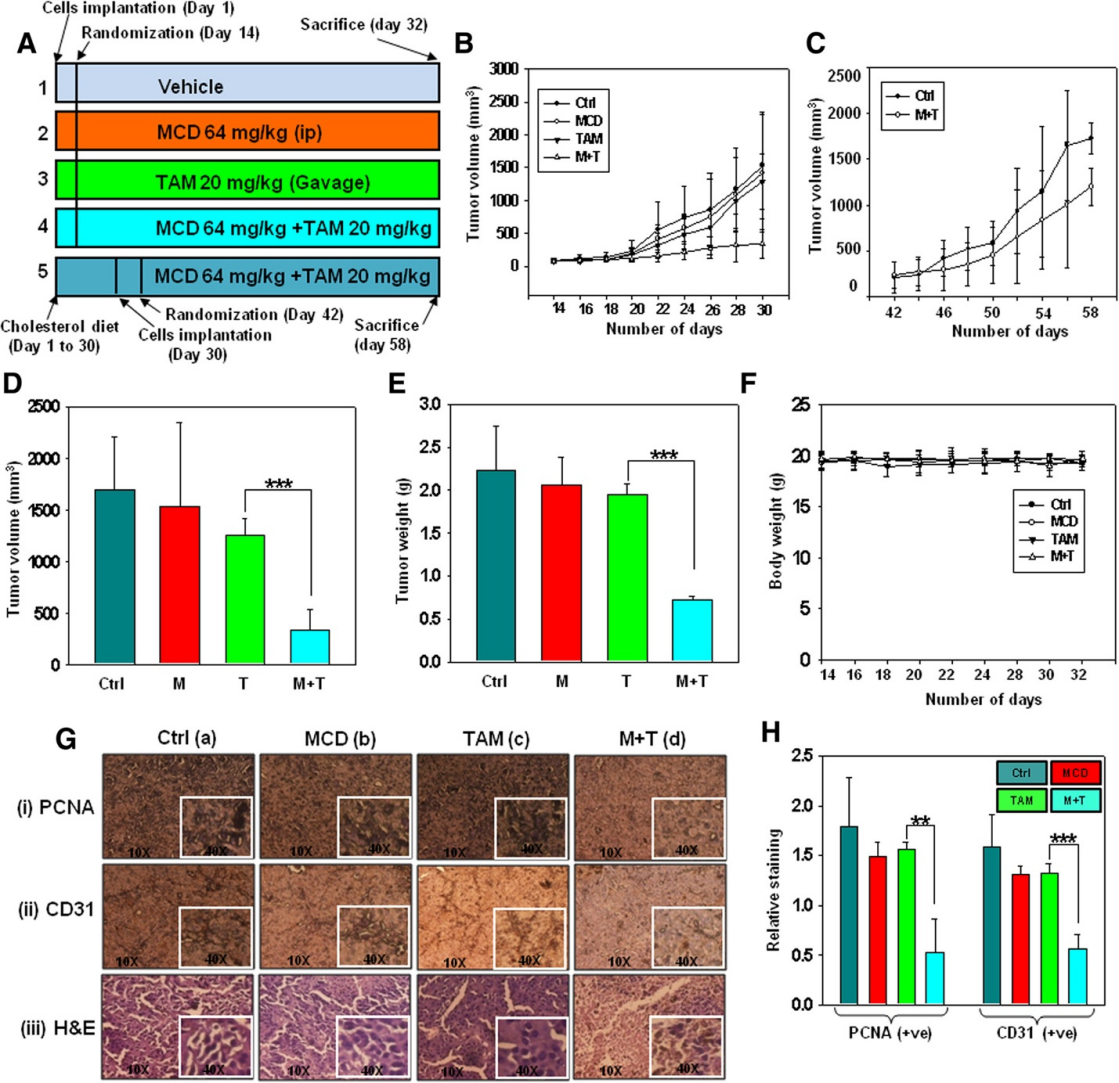
**Effect of tamoxifen and MCD on isografted mouse tumor model.** Mice were pre administered with 64 mg/kg of MCD (oral/alternative day) and 20 mg/kg of tamoxifen (i. p./alternative day). Control mice were administered with equal volume of normal saline on the same treatment day. **(A)** Experimental layout of *in vivo* study. **(B)** Tumor initiation and progression after drug administration in control and treated mice. **(C)** Changes in tumor volume in cholesterol supplemented mice after drug administration. **(D and E)** Bar graph (Mean ± SD) showing average tumor volume and tumor weight respectively of mice fed with normal diet, on the day of sacrifice (day 32). **(F)** Changes in body weight in mice during the course of treatments. **(G)** Representative panel of immunohistochemistry analysis of (i) PCNA, (ii) CD31 in the tumors of mice administered with MCD, tamoxifen and MCD along with tamoxifen. Magnification (10×) with inset at (40×).(iii) H&E staining of tumor of untreated and treated mice developed with DAB (brown) and counterstained with hematoxylin nuclei (blue). **(H)** Bar graph (Mean ± SD) showing quantitation of average number of PCNA positive cells selected from multiple fields by Image J software. (*P ≤ 0.05, **P ≤ 0.001, ***P ≤ 0.0001).

Having confirmed the enhanced effectiveness of tamoxifen in-combination with MCD *in vitro* in melanoma cells, we examined *in vivo* effects of this combination treatment against B16F10 cells isografted in C57BL/6J mice. After tumors of all the mice reached to an average volume of approximately 80 mm^3^, mice were randomly divided into four groups of normal diet and two groups of cholesterol supplemented diet. Animal experiments were performed according to layout (Figure 4A). Tumor volumes of mice were measured every alternate day. Tumors progressed very slowly in normal diet fed mice administered with tamoxifen and MCD combination as compared to either agent alone (Figure 4B). No significant reduction in tumor volume was detected in cholesterol supplemented diet fed mice administered with tamoxifen and MCD combination, and the tumor progression was similar to control mice (Figure 4C). The excised tumor size and tumor weight diminished by approximately 75% in normal diet fed mice administered with tamoxifen and MCD combination suggesting a synergistic effect of the combination (Figure 4D, E). In all the groups of mice administered with tamoxifen and MCD combination or either agent alone, body weight remained unaffected and no gross symptoms of toxicity or possible adverse side effects were detected upon visual inspection (Figure 4F). In order to investigate the precise mechanism of action of tamoxifen and MCD combination, the tumors were processed for immunohistochemical expression of PCNA (proliferation marker) and CD31 (angiogenesis marker). Diminished PCNA staining was detected in tumor sections of mice administered with tamoxifen and MCD combination as compared to untreated and either agent alone treated mice (Figure 4G (i) a-d). Angiogenesis is a critical factor for tumor growth and development and CD31 is widely used biomarker to highlight the degree of neoangiogenesis. CD31 expression is significantly reduced in tumor sections of mice administered with tamoxifen and MCD combination (Figure 4G (ii) a-d). The quantitative data of immunohistochemical analysis is depicted in Figure 4H. Furthermore, by hematoxylin and eosin staining, we found extensive necrotic areas in tumor sections of mice administered with tamoxifen and MCD combination compared to either agent alone (Figure 4G (iii) a-d). There were no apparent pathological abnormalities in the organs like liver, kidney, lung and heart of mice due to treatment with tamoxifen and MCD combination (Additional file 8: Figure S7). These results clearly suggest that antitumor effect of tamoxifen in- combination with MCD is a cumulative effect of increased antiproliferative and anti-angiogenesis activities.

### Mass spectrometric quantitation of tamoxifen in tumors and organs

**Figure 5:**
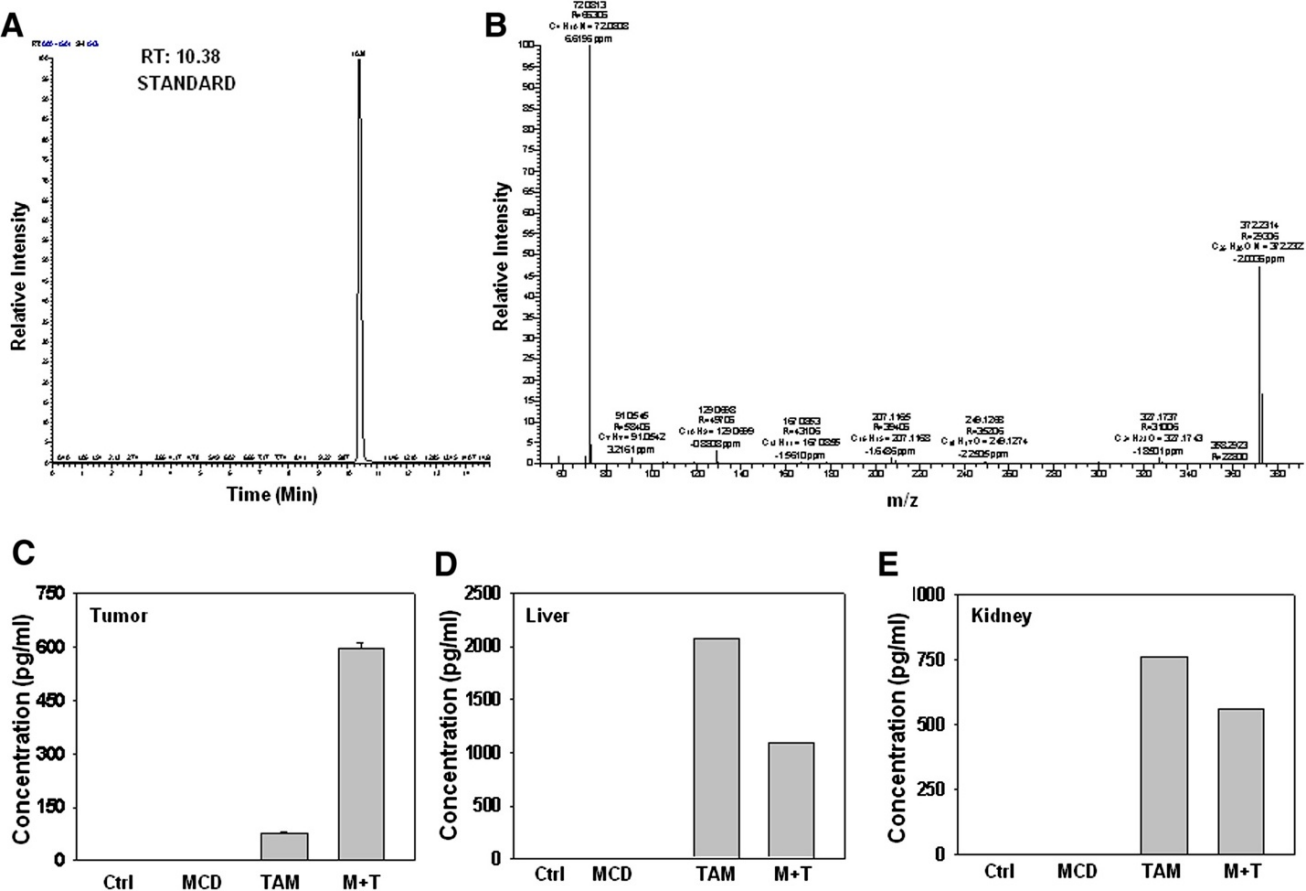
**Detection of tamoxifen in tumor of mice administered with tamoxifen and MCD. (A)** Extracted ion chromatogram (XIC) of selected fragment ion (m/z 72.0808) of tamoxifen. **(B)** MS/MS fragmentation pattern of tamoxifen. **(C)** Quantitation of tamoxifen by area under curve of tumor. **(D and E)** Quantitation of tamoxifen by area under curve of liver and kidney, respectively. Values of tumors are represented as mean ± SD (n = 3). For liver and kidney values are mean of two samples.

To investigate whether combination treatment enhances tamoxifen uptake, we performed mass spectrometry analysis in tumors, liver, kidney and serum samples of mice administered with tamoxifen and MCD combination and tamoxifen alone. Retention time (RT) for pure tamoxifen was determined to be 10.3 min (Figure 5A) and extracted ion chromatogram (XIC) of selected fragment ion was used to develop standard curve (Additional file 9: Figure S8). The fragmentation pattern of tamoxifen is shown in Figure 5B. Mass spectrometric analysis revealed presence of higher amount (7.2 fold) of tamoxifen in the tumors of mice administered with tamoxifen and MCD combination as compared to tamoxifen alone (Figure 5C). The level of tamoxifen in the liver and kidney of mice administered with tamoxifen and MCD combination was 30-50% lower than in mice administered with tamoxifen alone (Figure 5D, E). No detectable level of tamoxifen was detected in the plasma of mice from either treatment group (data no shown).

### Cav-1 level and Akt/ERK molecules facilitate methyl-β-cyclodextrin enhanced tamoxifen cytotoxic effects

**Figure 6:**
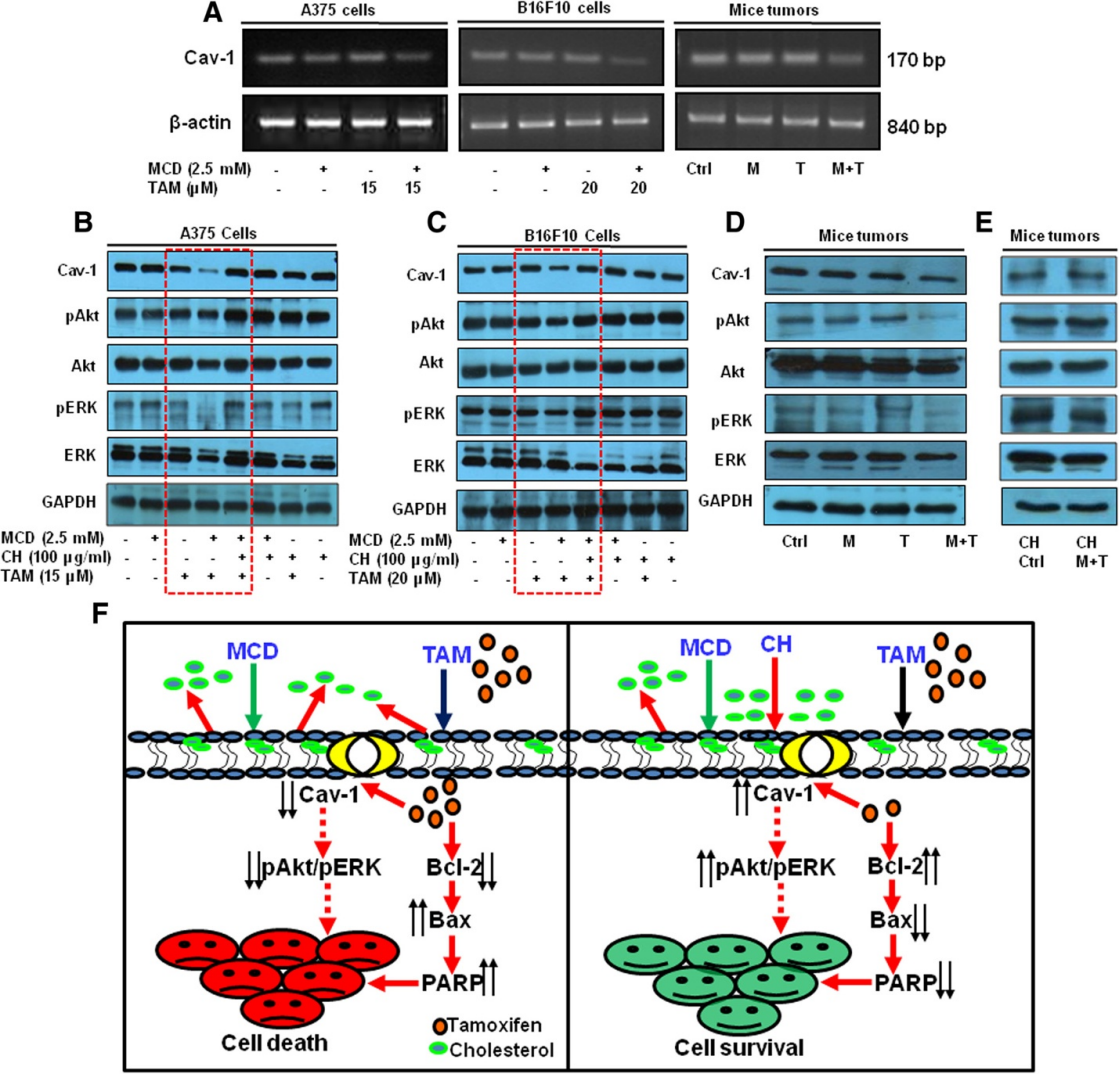
**MCD potentiates the effect of tamoxifen by altering Cav-1 levels and Akt/ERK signaling. (A)** Cav-1 mRNA levels were determined by semi-quantitative RT-PCR. A375, B16F10 cells and tumor from mice treated with indicated concentration of tamoxifen along with MCD, were processed for RT-PCR. β-actin was used as a loading control. **(B and C)** A375 and B16F10 cells, respectively, were treated with indicated concentration of tamoxifen in- combination with MCD and cholesterol, and whole cell lysate were subjected to Western blotting and expression level of Cav-1, pAkt, Akt, pERK and, ERK was assessed. **(D and E)** Tumor bearing mice were exposed to normal diet and cholesterol supplementation respectively, were treated with indicated dose of MCD and tamoxifen, and tumor lysates were subjected to Western blotting and expression level of Cav-1, pAkt, Akt, pERK and, ERK was assessed. GAPDH served as a loading control. **(F)** Schematic diagram of proposed mechanisms of MCD potentiating the effect of tamoxifen and its abrogation by cholesterol supplementation.

We have previously reported that MCD potentiates the effect of Carb and 5-FU by reducing activation of Akt and decreasing Bcl-2 level in breast cancer cells [21]. Also, association of Cav-1 and Akt/ERK signaling pathways with the mode of action of chemotherapeutic drugs has been reported earlier [24, 25]. In tumors lysates from normal diet fed mice administered with tamoxifen and MCD combination, Cav-1, pAkt and pERK levels were reduced (Figure 6D). Similarly, Cav-1, pAkt and pERK levels were also reduced in A375 and B16F10 cells treated with tamoxifen and MCD combination (Figure 6B, C respectively, highlighted by rectangular block). No changes in the levels of these proteins were detected in B16F1 cells treated with tamoxifen and MCD combination as compared to either agent alone (Additional file 3: Figure S2D). Additionally, decrease in Cav-1 mRNA was detected in A375 and B16F10 cells as well as tumors following treatment with tamoxifen and MCD combination (Figure 6A). Finally, we tested whether replenishment of membrane cholesterol by culturing cells in cholesterol rich medium would cause reversal of these alterations. Interestingly, cholesterol replacement prevents decrease in Cav-1, pAkt and pERK levels (Figure 6B, C respectively, highlighted by rectangular block) in the A375 and B16F10 cells as well as in tumors of mice fed with cholesterol and treated with tamoxifen and MCD combination (Figure 6E, F).

## Discussion

Due to accompanying drug resistance, effective therapeutic option to treat melanoma is still a medical challenge and nearly no improvement has been made for past thirty years. In this study, we investigated the growth inhibitory effect of tamoxifen in-combination with MCD *in vitro* and in isografted mice model of melanoma. We have demonstrated for the first time that only tamoxifen in-combination with MCD was effective in inhibiting the proliferation of melanoma cells regardless of Ras-Raf mutation status (Figure 1).

Tumor recurrence and metastases due to activating mutation in the mitogen-activated kinase (MAPK) pathway attributed to high mortality rate in melanoma patients. Mutation in MAPK enrolls enzyme Ras and Raf signalling cascade that leads to oncogenic cell proliferation and escape from apoptosis [4]. While human melanoma cell line (A375) carries BRAFV600E mutation and murine melanoma cell line (B16F10) accommodate distinct patterns of mutation in Ras gene with no active mutation in B-Raf oncogene [26]. Therefore, both human and murine melanoma cell lines were used to address the therapeutic outcome of tamoxifen in-combination with MCD. These mutational events are linked to activation of ERK and Akt and the high occurrence of deregulation pathways thus providing a rationale for the development of target based chemotherapeutics for the treatment of melanoma [27]. Although, medical fraternity aims to formulate therapeutic strategies toward targeting mutated B-Raf/N-Ras, its downstream molecules ERK and Akt have not been tested [28, 29]. Recently, the introductions of BRAF inhibitors and new immunotherapies have provided more efficient treatment options with negligible toxicity [30].

Studies have shown that various signalling molecules enriched on the lipid rafts of plasma membrane are associated with a number of biological processes and their disruption impairs these signaling events [31, 32]. Hence, various components of plasma membrane have been a promising target for cancer chemotherapy. Cholesterol, an important component of lipid rafts, maintains the stability and architecture of cell membrane. Its accumulation has been reported in cancers such as prostate and oral cancer [33, 34]. Also, cholesterol metabolism is highly dysregulated in cancers including myeloid leukemia and breast cancer [35, 36]. Amount of cholesterol present in the lipid rafts of cell membrane influences trafficking of drugs and other molecules through diffusion or receptor meditated uptake [17]. In these contexts, cholesterol depleting agents, cyclodextrins, are gaining importance in pharmaceutical industries because of their effectiveness in enhancing the bioavailability and solubility of drugs [37, 38]. MCD is a frequently used FDA approved cyclodextrin to disrupt lipid raft. Depletion of cholesterol from the plasma membrane by MCD leads to modification of membrane permeability thereby altering the signalling and the transport of many molecules in cells [39–42]. Also, it is been reported to cause apoptosis and caveolae internalization in addition to abrogation of Akt signaling in human epidermoid carcinoma cells [43]. Present study provides evidences that depletion of cholesterol from the membrane sensitizes cells towards tamoxifen mediated cell death through down regulation of Cav-1 and reduced phosphorylation of Akt/ERK. Additionally, we show that MCD not only potentiated the cytotoxic effects of tamoxifen in melanoma cells *in vitro* (Figure 1) but also in B16F10 isografted mice model (Figure 4).

Tamoxifen alone or in-combination with other chemotherapeutic drugs has been reported to show poor response rate in the treatment of melanoma [15, 16]. In this study, MCD was used as a tool to enhance the cytotoxic effect of tamoxifen, and we found that this combination treatment synergistically inhibited the proliferation of melanoma cells. *In vitro* combination of tamoxifen and MCD efficiently induced DNA fragmentation and arrest in G1 phase of cell cycle (Figure 2) in A375 and B16F10 cells. *In vivo*, we demonstrated that combination of tamoxifen (20 mg/kg) and MCD (64 mg/kg) significantly suppresses the tumor growth of mice as compared to either agent alone with no apparent body weight loss and toxicity to vital organs. The reduction in tumor size is primarily due to enhanced antiproliferative and antiangiogenic effect of combination treatment. Our results are in agreement to the previous report wherein the decrease in the expression of PCNA and CD31 has been correlated with reduction of tumor size and overall disease free survival [44]. Quantitation of tamoxifen in tumor samples by mass spectrometry highlights the fact that reduction of tumor size was essentially achieved because of increase in tamoxifen levels in the tumor of mice administered with tamoxifen and MCD combintaion as compared to tamoxifen alone (Figure 5C). Concomitantly, the increased drug uptake specifically in tumor reduces the accumulation of tamoxifen in liver and kidney and thus it is likely to be less toxic to the organs, which is a major concern of chemotherapy (Figure 5D, E).

Akt and ERK are major regulators of cell survival pathways governed by growth factors and various cytokines [45]. Activation of PI3K/Akt or ERK pathways are associated with cell differentiation, cell proliferation and resistance to apoptosis in various cancers including melanoma [46–48]. Cav-1 is an important player for the regulation of cellular cholesterol homeostasis, a process that controls the accumulation of cholesterol on cell membrane. In Cav-1 knock-out mice, depleted level of free cholesterol on the surface of mouse embryonic fibroblast and mouse peritoneal macrophages has been reported [49]. In an earlier study, we have reported a positive correlation between Cav-1 levels to rapid progression of melanoma in mice fed with high fat diet [50]. Cav-1 is known to exert direct or indirect effect on the activation of Akt and ERK pathways [24, 25]. Thus, it is likely that decrease in Cav-1 levels acts as an upstream event in deactivation of intrinsic Akt and ERK growth stimulatory signals in highly metastatic melanoma cells (A375 and B16F10) whereas in non-metastatic melanoma cells (B16F1) no such effect was detected (Figure 6B, C, Additional file 3: Figure S2D respectively). Our findings indicate that cholesterol supplementation prevented MCD potentiated tamoxifen cell death *in vitro* as well as *in vivo* (Figures 3 and 4) by reversing the molecular alterations as shown in schematic model (Figure 6F).

## Conclusions

The present findings emphasize the fact that cholesterol depleting agent, MCD sensitizes highly metastatic melanoma cells towards tamoxifen treatment. Combinatorial treatment of tamoxifen and MCD induces cytotoxicity by causing cell cycle arrest, induction of apoptosis and decreases tumor growth *in vivo*. Taken together, our results strongly provide the evidence that tamoxifen in- combination with MCD could have potential implications in melanoma treatment as well as other solid tumors.

## Material and methods

### Drugs, chemicals and antibodies

Tamoxifen (TAM), methyl β-cyclodextrin (MCD), cholesterol (CH) and methylthioazole-tetrazolium (MTT) were purchased from Sigma-Aldrich (Sigma Aldrich, MO). TAM and MCD were dissolved in ethanol and water respectively to prepare 100 mM stock and further diluted in culture medium. Antibodies against pAkt, Akt, pERK, ERK, Cav-1, PARP, Bax, Bcl-2, Cdk4, Cdk1/2, cyclin D1, pRb, Rb, ERα, ERβ and GAPDH were purchased from Santa Cruz Biotechnology (Santa Cruz, CA).

### Cell culture conditions

A375 (human melanoma), B16F10 (murine melanoma) and B16F1 (non metastatic melanoma), HeLa (cervical) and MCF-7 (breast) cancer cells were purchased from American Type Culture Collection (ATCC) Manassas, VA and maintained in our in-house Cell Repository. Cells were cultured in Dulbecco’s modified Eagle’s medium (DMEM) with 10% heat inactivated FBS (Hyclone, UT), Penicillin (100U/ml), Streptomycin (100 μg/ml) (Invitrogen Life Technologies, CA) and incubated at 37°C in 5% CO_2_ incubator (Thermo Scientific, NC).

### Mode of MCD and tamoxifen combination treatment

Cells were pre-exposed to MCD for 4 h. Thereafter, cells were washed with fresh medium and then medium containing indicated concentrations of tamoxifen was added for 24 h. For cholesterol (CH) treatment, cells were incubated with MCD for 4 h, washed thereafter and fresh medium containing cholesterol (100 μg/ml) was added. Four hours later, tamoxifen was added and cells were incubated for further 24 h.

### MTT (methylthioazole tetrazolium) assay

Cells (5 × 10^3^/well) were plated in 96-well plates and allowed to adhere for 24 h at 37°C. Next day, cells were treated with varying concentration of tamoxifen for 24 h with or without MCD or cholesterol as described earlier in mode of treatment. Viability of cells was measured by MTT assay as described [50]. Any synergistic effect resulting from combination of drugs was calculated by coefficient of drug interaction (CDI) as follows:


AB is the ratio of the combination of drug groups to control group; A or B is the ratio of the single drug group to control group. The CDI value > 1: antagonistic effect, CDI = 1: additive and CDI < 1: synergistic effect [51].

### Whole cell lysate preparation and western blotting

Whole cell lysates were prepared and immunoblotting was performed as described previously [50].

### Long term clonogenic survival assay

Cells (5 × 10^3^/well) were plated in 12-well plates and allowed to adhere for 24 h. Cells were treated as per experimental requirement. After 24 h of treatment medium was replaced with fresh medium and cells were allowed to grow for 10–15 days. After completion of experiment, surviving cells were washed with PBS and fixed with chilled 3% paraformaldehyde. The surviving cells were stained with 0.05% crystal violet dye and images were captured by camera (Olympus, Tokyo, Japan). The number of colonies was counted by using Image J software.

### RNA extraction, cDNA synthesis and quantitative RT-PCR

Cells were treated with MCD and tamoxifen as described earlier. Also, tumor sections of mice were stored at -80°C in TRIzol reagent (Invitrogen, CA) after excision until processing for RT-PCR. Total RNA from the cells and tumors was extracted as per the manufacturer’s instructions (TRIzol reagent Invitrogen, CA). cDNA synthesis and RT-PCR were performed as described earlier [24]. Primer pairs used are as follows: Cav-1 5′-AGA CTCGGAGGGACATCTCTACAC-3′ (F), 5′-ACTGTGTGTCCCTTCTGGTTCTG-3;(R) and for β-Actin 5′-ATCTGGCACCACACCTTCTACAATGAGCTGCG-3′ (F), 5′-CGTCATACTCCTGCTTGCTGATCCACATCTGC-3′ (R). The annealing temperature used for both Cav-1 and β-actin was 58°C.

### Cholesterol estimation

Cells (3 × 10^5^) were plated in 35 mm culture dish and treated with MCD, cholesterol and tamoxifen as described earlier. Cells were lysed in PBS containing 2% Triton X-100 for 10 min. After centrifugation (12,000 rpm, 15 min), resulting supernatant was used for cholesterol estimation. For cholesterol release assay, medium was collected and concentrated by using Speed Vac (Thermo Savant, MA). In animal experiments blood was collected from mice fed with 2% cholesterol by approved tail cap method. Cholesterol was estimated by using kit with sensitivity range of 1 mg/dL–750 mg/dL purchased from Spinreact (Girona, Spain) as per the manufacturer’s instructions.

### LDH release assay

Cells (3 × 10^5^) were plated in 35 mm culture dish and treated as described earlier, medium was collected and LDH release was measured according to manufacturer’s protocol using LDH activity assay kit (Spinreact, Girona, Spain).

### Cell cycle analysis

Cells (3 × 10^5^) were plated in 35 mm culture dish and treated with 2.5 mM of MCD for 4 h, washed twice with fresh medium and further grown in culture medium for additional 12 h. In experiments related to MCD and tamoxifen, cells were treated with both the drugs as described earlier. Cells were collected and processed for cell cycle analysis as described elsewhere [52].

### Agarose gel electrophoresis

Cells (3 × 10^5^) were plated in 35 mm culture dish and treated with MCD and tamoxifen as described earlier and agarose gel electrophoresis and LDH release assay performed as described elsewhere [53].

### Mass spectrometric analysis

Tamoxifen was extracted from tumors, plasma of tumor bearing mice and from liver, kidney of mice injected with MCD and tamoxifen combination according to described method [54]. Tamoxifen was quantified in biological samples in duplicate by selected reaction monitoring using hybrid quadrupole Orbitrap mass analyzer (Q-Exactive, Thermo Scientific, Germany) in a high resolution mode. Briefly, 5 μL of the final extract was injected to Accela UPLC (Thermo Scientific, Germany) and the separation was performed using a reverse phase Hypersil Gold C18 5 μm column (150 × 4.6 mm) with a flow rate of 500 μL/min of binary solvent system consisting of mobile phase A: water with 0.1% formic acid and mobile phase B: acetonitrile with 0.1% formic acid. The mass spectra were acquired in high resolution (70000) mode by using Xcalibur and data was processed by Quant software (Thermo Scientific, Germany). The method consisted of full scans and targeted MS/MS of selected precursor ion at a defined mass and retention time according to described settings [55]. The standard curve was developed by plotting the log_10_ value of area under curve (AUC) of the selected fragment of tamoxifen (m/z 72.0808) by extracted ion chromatogram (XIC) against log_10_ value of serial dilutions of pure tamoxifen ranging from 50 fg to 500 ng. Tamoxifen was identified and quantified by comparing the XIC of selected fragment ion (m/z 72.0808).

### *In vivo*experiments

All animal experiments were performed according to the Institutional guidelines, following a protocol approved by the Institutional Animal Ethic Committee (IAEC). Five to six weeks old male C57BL/6J (weight 20 ± 2 g) were acquired from experimental animal facility (EAF) of National Centre for Cell Science, Pune, India. B16F10 cells (1 × 10^6^/mice) were injected subcutaneously on the right flank of each mouse. After 12–14 days, palpable tumor bearing mice were randomly divided into four groups (n = 6). Group (a), mice administered with vehicle control, group (b), mice administered with MCD (64 mg/kg, intraperitoneally), group (c), mice administered with tamoxifen (20 mg/kg, orally) and group (d), mice administered with MCD and tamoxifen. MCD and tamoxifen were dissolved in sterile water and ethanol respectively and further diluted with PBS. In cholesterol feeding experiment, mice were fed with chow containing 2% cholesterol for 30 days and total serum cholesterol was measured every 15 days. Subsequently, mice were divided into two groups. Group one referred as control group whereas group two mice were administered with MCD and tamoxifen. Both the groups of mice were fed with cholesterol during the course of experiment. At the end of experiment, mice were sacrificed by cervical dislocation and tumors excised. Size of tumors during the course of experiment was measured using caliper in two dimensions. Tumor volume (mm^3^) was calculated according to the formula AXB^2^X0.52 (A, length; B, width; all parameters in millimeters). For immunohistochemical and histopathological studies, sections of tumors and organs were fixed into 10% paraformaldehyde immediately after excision. Remaining part of tumors was stored at -80°C for RT-PCR and lysate preparation used for Western blotting.

### Immunohistochemical and histopathological studies

Fine sections (4 μm) were prepared from formalin fixed paraffin embedded tumor tissue and fixed on glass slides (Safeline Histopathology, Pune, India). For immunohistochemistry, slides were deparaffinized by xylene solution twice for 10 min and subsequently dehydrated in graded alcohol (100%, 95%, 70% and 50%). Endogenous peroxidase activity was blocked by 0.01% H_2_O_2._ For antigen retrieval, slides were boiled in sodium citrate buffer (0.01 M, pH 4.5) at 100°C for 10 min and allowed to cool at room temperature. BSA (0.2%) was used for blocking for 1 h. After washing with TBST, slides were probed with CD31 and PCNA antibodies specific for IHC (Santa Cruz Biotechnology, CA) and incubated at 4°C overnight. Slides were washed with TBST and probed with compatible HRP-conjugated secondary antibody for 3 h. Slides were stained with diaminobenzidine (DAB) for 10 min followed by counterstaining with hematoxylin and eosin. Slides were mounted and analysis of indicated proteins was performed. For histopathology, deparaffinized slides were stained with hematoxylin and eosin and microscopic analysis for cell density, cellular morphology and necrosis was performed and images were captured by DP71 camera attached with microscope (Olympus, Tokyo, Japan). The staining of cells was quantified by Image J software.

### Statistical analysis

Statistical analysis and data comparison were performed by Student’s 2-tailed unpaired t-test by using Sigma Plot software (Systat Software Inc, CA). The values of P <0.05 were considered statistically significant. Quantitation of colonies and relative staining of PCNA of cells were done by using NIH Image J software (Image J Freeware; http://rsb.info.nih.gov/ij/).

## Electronic supplementary material

Additional file 1: Table S1: IC_50_ values of tamoxifen for A375, B16F10 and B16F1 cells. **Table S2.** Drug interaction was analyzed by calculating coefficient of drug interaction (CDI) as described in materials and method section. (TIFF 964 KB)

Additional file 2: Figure S1: Low-dose treatment of MCD does not induce toxicity in A375 and B16F10 cells. (A and D) Cells were treated with indicated concentration of tamoxifen and MCD and cells were subjected to MTT assay. (B and E) Display of different phases of cell cycle represented as percent cell population. (C and F) LDH release assay. Bar graph represents the mean ± SD of an experiment done in triplicate. (*P ≤ 0.05, **P ≤ 0.001, ***P ≤ 0.0001). (TIFF 1 MB)

Additional file 3: Figure S2: Tamoxifen and MCD combination treatment does not affect survival of B16F1 cells (non-metastatic). (A) Cells were treated with indicated concentration of MCD for 1 and 4 h, (B) Cells were treated with indicated concentration of tamoxifen and MCD for 24 h and cells were subjected to MTT assay. (C) Clonogenic survival assay. (D) Representative Western blots showing protein level of indicated molecules. In MTT assay, bar graph represents the mean ± SD of an experiment done in triplicate. (TIFF 184 KB)

Additional file 4: Figure S3: MCD does not affect survival of A375 and B16F10 cells treated with various chemotherapeutic drugs. (A-C) A375, (D-F) B16F10 cells were treated with indicated concentration of MCD followed by treatment with either of carboplatin (Carb), doxorubicin (DOX) or 5-flurouracil (5-FU) for further 24 h and cells were subjected to MTT assay. Bar graph represents the mean ± SD of an experiment done in triplicate. (*P ≤ 0.05, **P ≤ 0.001, ***P ≤ 0.0001). (TIFF 1 MB)

Additional file 5: Figure S4: MCD potentiates cell toxicity of higher doses of DTIC to melanoma cells. (A and C) A375 and B16F10 cells were treated with indicated concentration of MCD and DTIC for 24 h, and cells were subjected to MTT assay. (C and D) Clonogenic survival assay. Bar graph represents the mean ± SD of an experiment done in triplicate. (TIFF 1 MB)

Additional file 6: Figure S5: MCD enhances the susceptibility of melanoma cells to tamoxifen by altering cell cycle regulatory molecules. A375 and B16F10 cells were treated with indicated concentration of tamoxifen and MCD. Cell lysates were prepared and proteins were resolved on 10-12% SDS-PAGE and processed for Western blotting analysis. (A-D) Representative Western blots showing protein level of indicated cell cycle regulatory molecules. (TIFF 1 MB)

Additional file 7: Figure S6: Total cholesterol (CH) estimation in cells and in spent medium owing to the drug treatment. Cells were treated with indicated concentration of tamoxifen and MCD and cholesterol was estimated in whole cell extract (A and D) and in culture medium (C and F). (B and E) cells were treated with indicated concentration of MCD, tamoxifen as well cholesterol, level of cholesterol was estimated in cell lysate. Bar graph represents the mean ± SD of an experiment done in triplicate (*P ≤ 0.05, **P ≤ 0.001, ***P ≤ 0.0001). (TIFF 2 MB)

Additional file 8: Figure S7: Histopathological analysis of major vital organs. Liver, kidney, lungs and heart tissues were fixed in 4% formaldehyde. The processed tissues sections were stained by hematoxylin and eosin (H&E) (magnification, ×400; scale bars, 100 μm). (TIFF 7 MB)

Additional file 9: Figure S8: HPLC profile of standard curve of different concentration of tamoxifen. Standard curve of tamoxifen was generated by plotting log10 (AUC) Vs log10 (concentration of tamoxifen). (TIFF 1 MB)

## Abbreviations

MCD: Methyl β-cyclodextrin
TAM: Tamoxifen
CH: Cholesterol
LDH: Lactate dehydrogenase
Cav-1: Caveolin-1
Carb: Carboplatin
DOX: Doxorubicin
5-FU: 5- Fluorouracil
DTIC: Dacarbazine
CDI: Coefficient of drug interaction
DAB: Diaminobenzidine
H&E: Hematoxylin and eosin
XIC: Extracted ion chromatogram.
