# Supplementary figures and images for: Cholesterol depletion by methyl-β-cyclodextrin augments tamoxifen induced cell death by enhancing its uptake in melanoma
Source: Mol Cancer. 2014 Sep 1;13:204. doi: 10.1186/1476-4598-13-204 (PMC4175626; doi:10.1186/1476-4598-13-204)

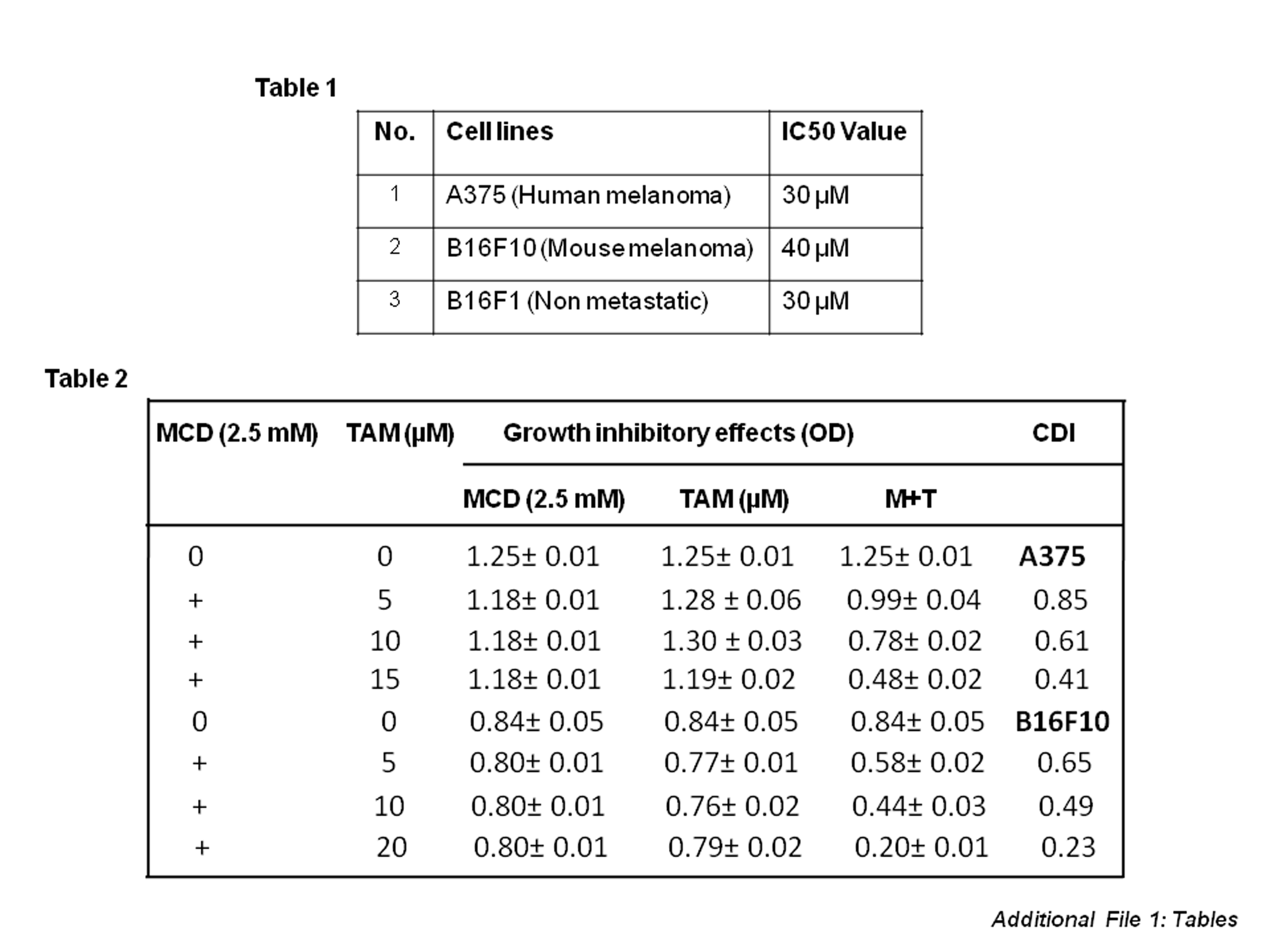

Supplement: Supplementary file 1 — Additional file 1: Table S1: IC50 values of tamoxifen for A375, B16F10 and B16F1 cells. Table S2. Drug interaction was analyzed by calculating coefficient of drug interaction (CDI) as described in materials and method section. (TIFF 964 KB) [file 12943_2014_1414_MOESM1_ESM.tiff]

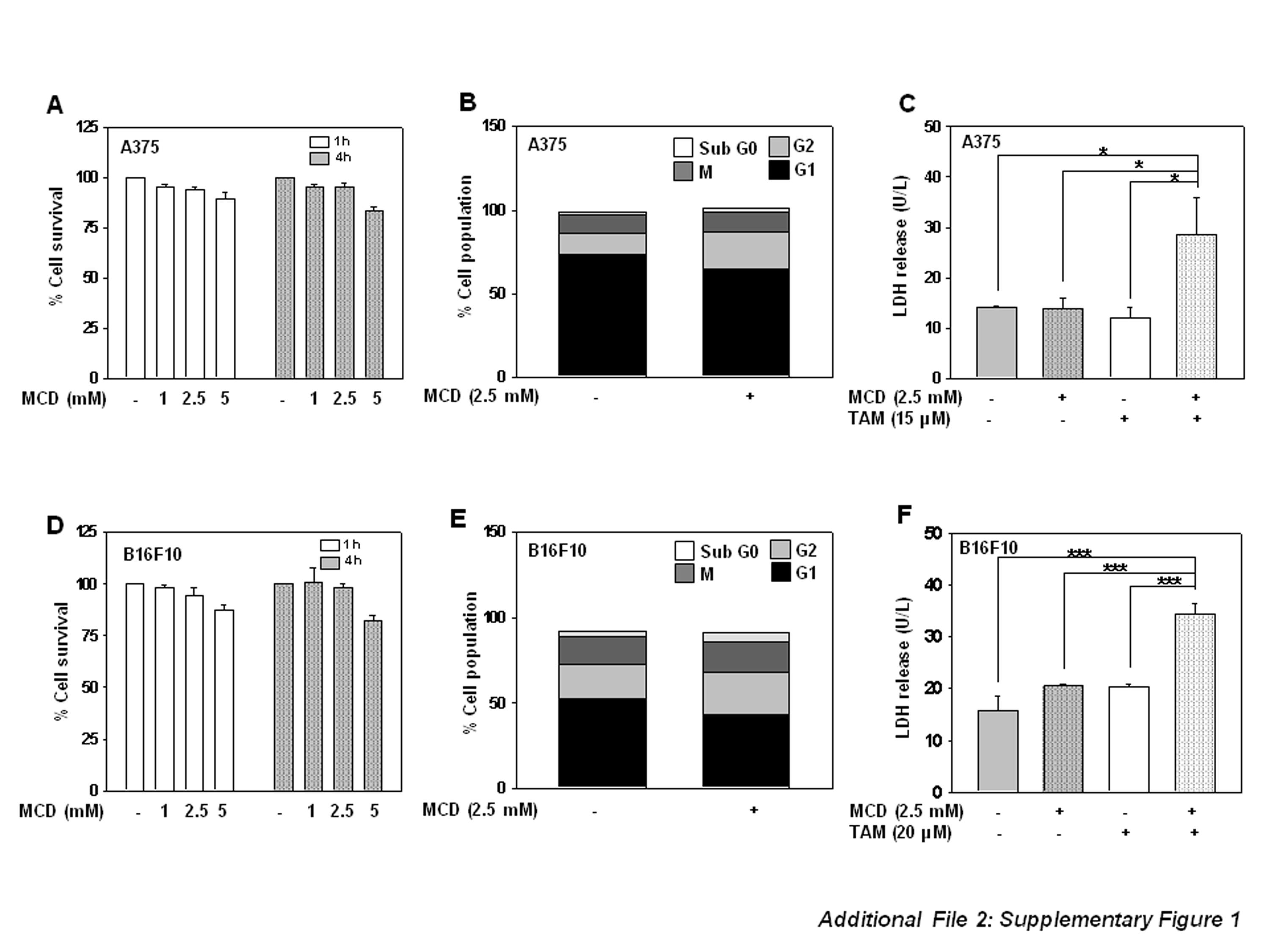

Supplement: Supplementary file 2 — Additional file 2: Figure S1: Low-dose treatment of MCD does not induce toxicity in A375 and B16F10 cells. (A and D) Cells were treated with indicated concentration of tamoxifen and MCD and cells were subjected to MTT assay. (B and E) Display of different phases of cell cycle represented as percent cell population. (C and F) LDH release assay. Bar graph represents the mean ± SD of an experiment done in triplicate. (*P ≤ 0.05, **P ≤ 0.001, ***P ≤ 0.0001). (TIFF 1 MB) [file 12943_2014_1414_MOESM2_ESM.tiff]

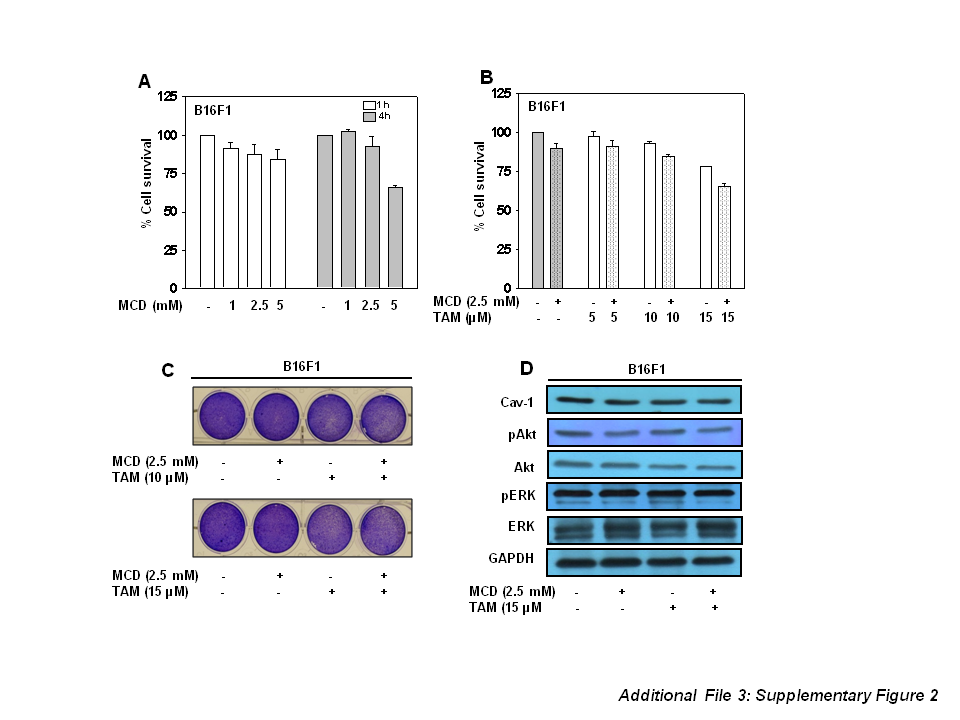

Supplement: Supplementary file 3 — Additional file 3: Figure S2: Tamoxifen and MCD combination treatment does not affect survival of B16F1 cells (non-metastatic). (A) Cells were treated with indicated concentration of MCD for 1 and 4 h, (B) Cells were treated with indicated concentration of tamoxifen and MCD for 24 h and cells were subjected to MTT assay. (C) Clonogenic survival assay. (D) Representative Western blots showing protein level of indicated molecules. In MTT assay, bar graph represents the mean ± SD of an experiment done in triplicate. (TIFF 184 KB) [file 12943_2014_1414_MOESM3_ESM.tiff]

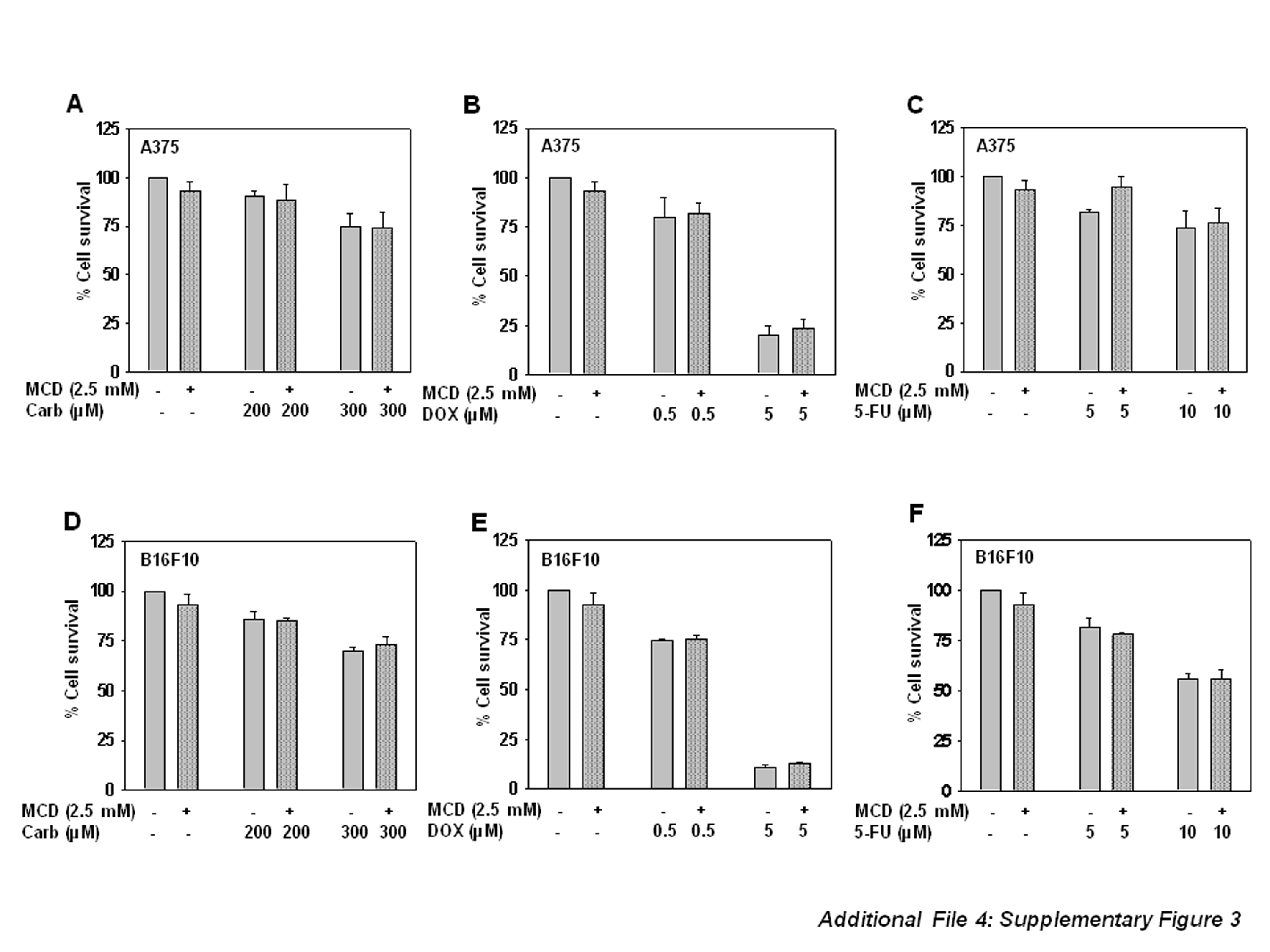

Supplement: Supplementary file 4 — Additional file 4: Figure S3: MCD does not affect survival of A375 and B16F10 cells treated with various chemotherapeutic drugs. (A-C) A375, (D-F) B16F10 cells were treated with indicated concentration of MCD followed by treatment with either of carboplatin (Carb), doxorubicin (DOX) or 5-flurouracil (5-FU) for further 24 h and cells were subjected to MTT assay. Bar graph represents the mean ± SD of an experiment done in triplicate. (*P ≤ 0.05, **P ≤ 0.001, ***P ≤ 0.0001). (TIFF 1 MB) [file 12943_2014_1414_MOESM4_ESM.tiff]

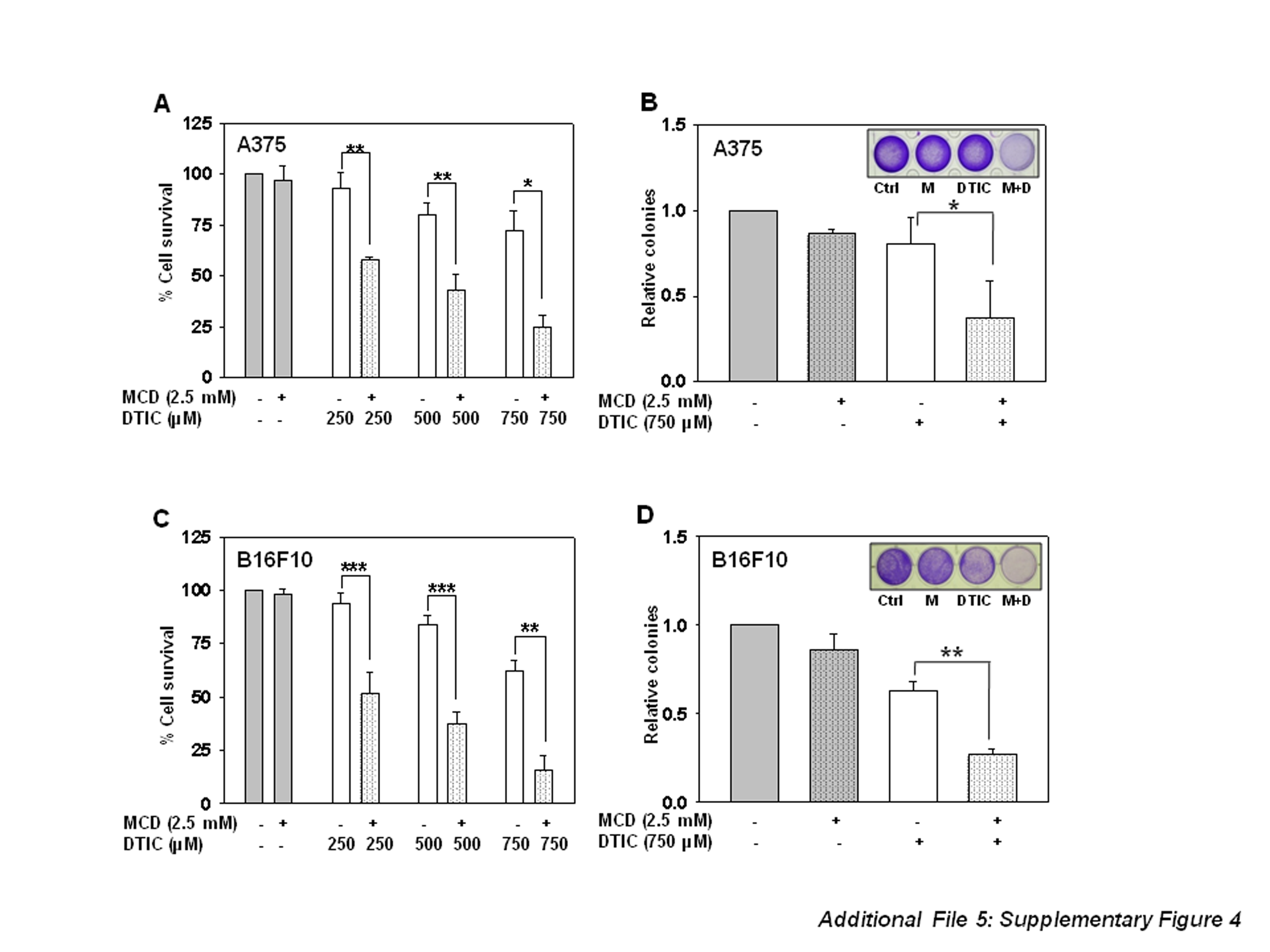

Supplement: Supplementary file 5 — Additional file 5: Figure S4: MCD potentiates cell toxicity of higher doses of DTIC to melanoma cells. (A and C) A375 and B16F10 cells were treated with indicated concentration of MCD and DTIC for 24 h, and cells were subjected to MTT assay. (C and D) Clonogenic survival assay. Bar graph represents the mean ± SD of an experiment done in triplicate. (TIFF 1 MB) [file 12943_2014_1414_MOESM5_ESM.tiff]

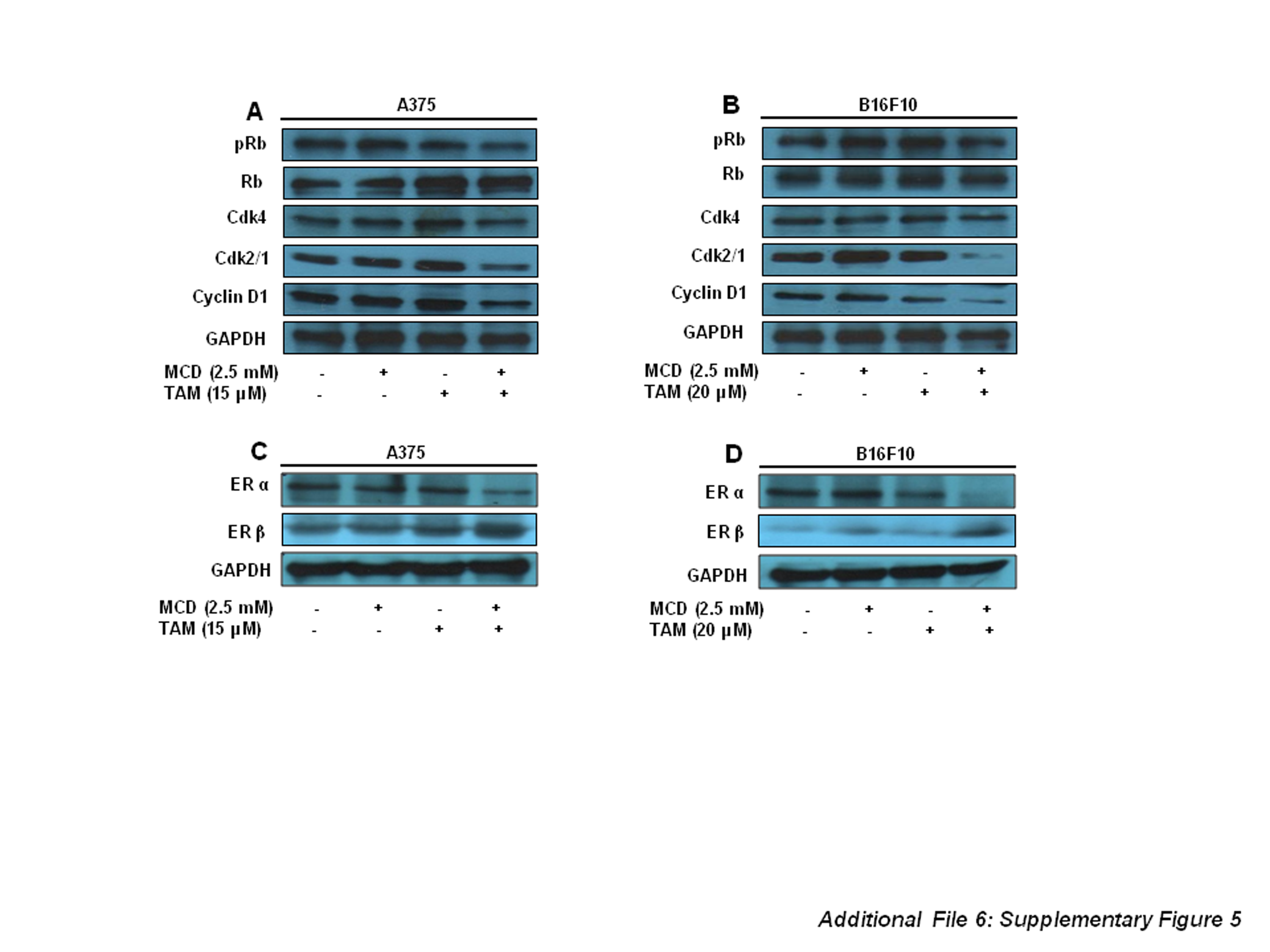

Supplement: Supplementary file 6 — Additional file 6: Figure S5: MCD enhances the susceptibility of melanoma cells to tamoxifen by altering cell cycle regulatory molecules. A375 and B16F10 cells were treated with indicated concentration of tamoxifen and MCD. Cell lysates were prepared and proteins were resolved on 10-12% SDS-PAGE and processed for Western blotting analysis. (A-D) Representative Western blots showing protein level of indicated cell cycle regulatory molecules. (TIFF 1 MB) [file 12943_2014_1414_MOESM6_ESM.tiff]

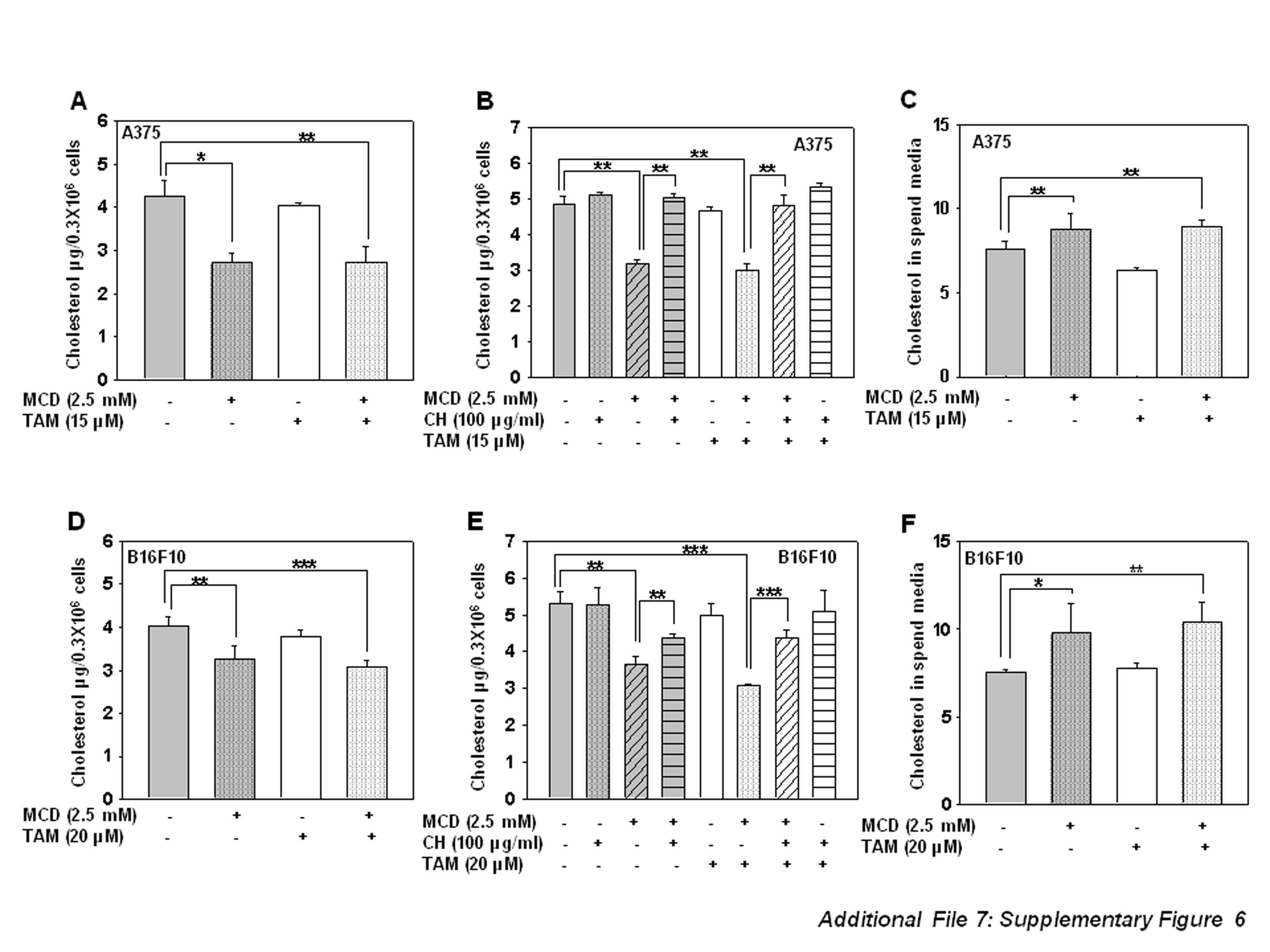

Supplement: Supplementary file 7 — Additional file 7: Figure S6: Total cholesterol (CH) estimation in cells and in spent medium owing to the drug treatment. Cells were treated with indicated concentration of tamoxifen and MCD and cholesterol was estimated in whole cell extract (A and D) and in culture medium (C and F). (B and E) cells were treated with indicated concentration of MCD, tamoxifen as well cholesterol, level of cholesterol was estimated in cell lysate. Bar graph represents the mean ± SD of an experiment done in triplicate (*P ≤ 0.05, **P ≤ 0.001, ***P ≤ 0.0001). (TIFF 2 MB) [file 12943_2014_1414_MOESM7_ESM.tiff]

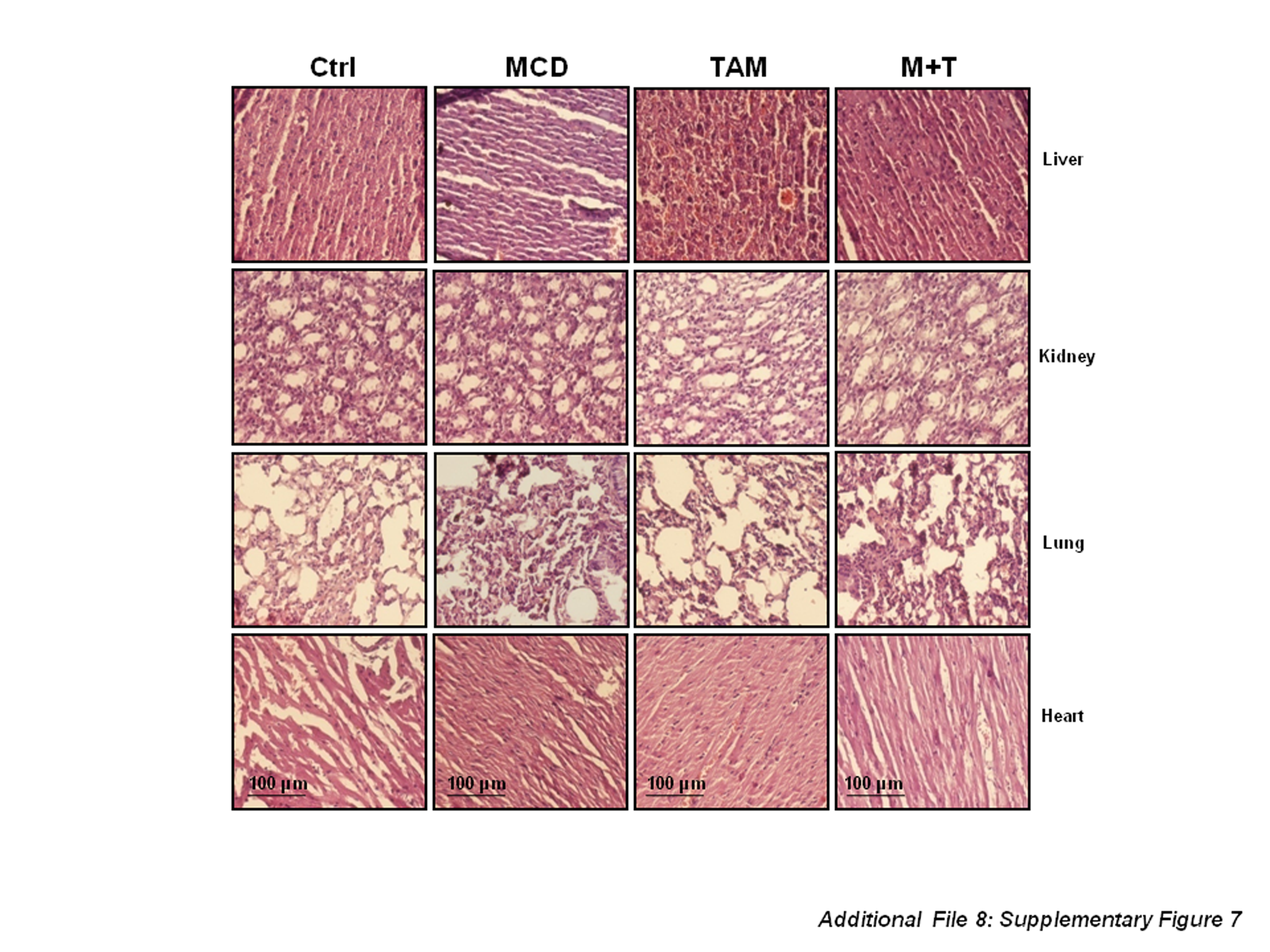

Supplement: Supplementary file 8 — Additional file 8: Figure S7: Histopathological analysis of major vital organs. Liver, kidney, lungs and heart tissues were fixed in 4% formaldehyde. The processed tissues sections were stained by hematoxylin and eosin (H&E) (magnification, ×400; scale bars, 100 μm). (TIFF 7 MB) [file 12943_2014_1414_MOESM8_ESM.tiff]

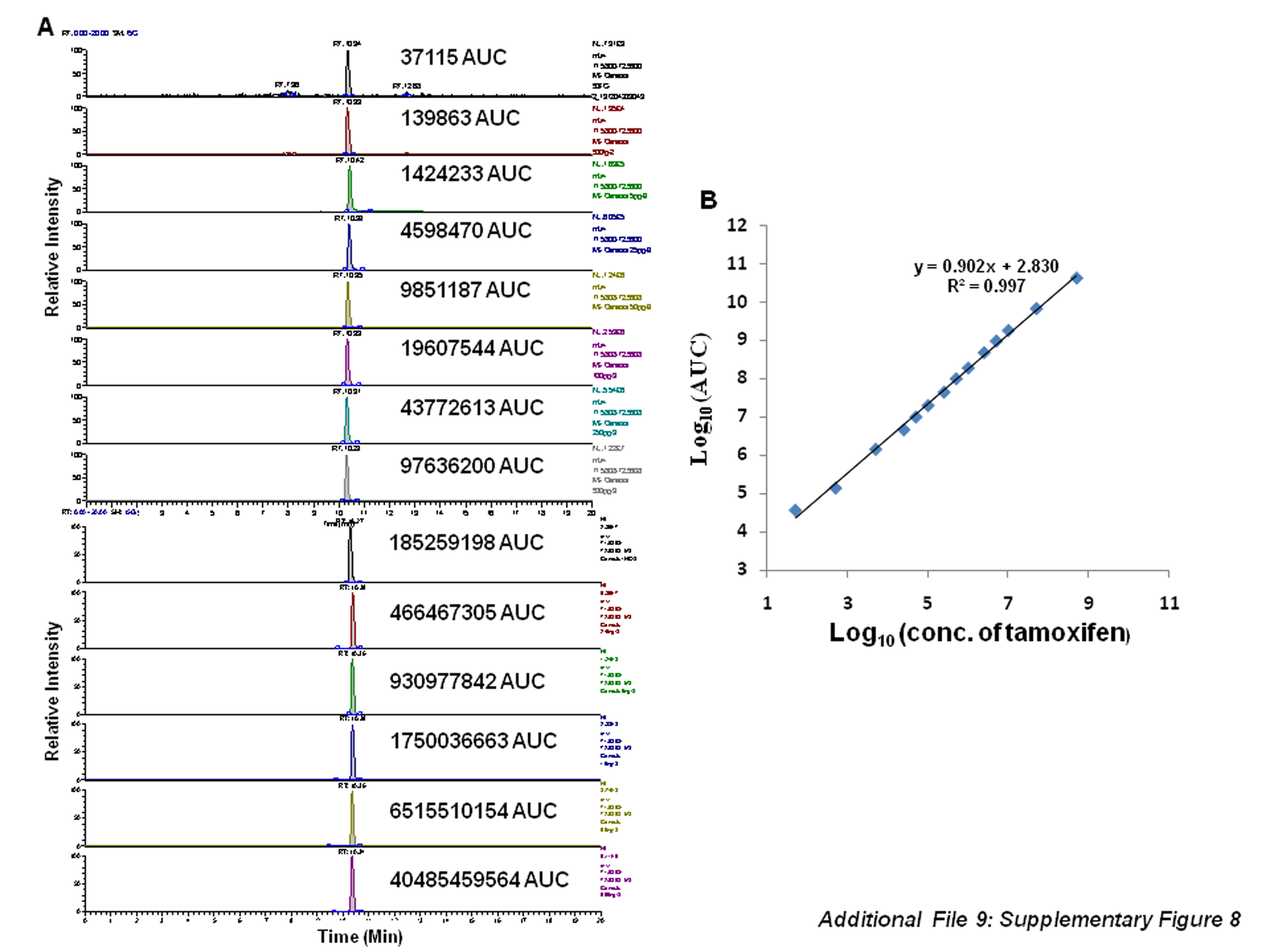

Supplement: Supplementary file 9 — Additional file 9: Figure S8: HPLC profile of standard curve of different concentration of tamoxifen. Standard curve of tamoxifen was generated by plotting log10 (AUC) Vs log10 (concentration of tamoxifen). (TIFF 1 MB) [file 12943_2014_1414_MOESM9_ESM.tiff]
